# WNK1/HSN2 mediates neurite outgrowth and differentiation via a OSR1/GSK3β-LHX8 pathway

**DOI:** 10.1038/s41598-022-20271-y

**Published:** 2022-09-23

**Authors:** Masahiro Shimizu, Hiroshi Shibuya

**Affiliations:** Department of Molecular Cell Biology, Medical Research Institute, Tokyo Medical and Dental University (TMDU), Bunkyo-ku, Tokyo, 113-8510 Japan

**Keywords:** Biochemistry, Cell biology, Molecular biology

## Abstract

With no lysine kinase 1 (WNK1) phosphorylates and activates STE20/SPS1-related proline-alanine-rich protein kinase (SPAK) and oxidative stress responsive kinase 1 (OSR1) to regulate ion homeostasis in the kidney. Mutations in *WNK1* result in dysregulation of the WNK1-SPAK/OSR1 pathway and cause pseudohypoaldosteronism type II (PHAII), a form of hypertension. WNK1 is also involved in the autosomal recessive neuropathy, hereditary sensory and autonomic neuropathy type II (HSANII). Mutations in a neural-specific splice variant of *WNK1* (HSN2) cause HSANII. However, the mechanisms underlying HSN2 regulation in neurons and effects of HSN2 mutants remain unclear. Here, we found that HSN2 regulated neurite outgrowth through OSR1 activation and glycogen synthase kinase 3β (GSK3β). Moreover, HSN2-OSR1 and HSN2-GSK3β signalling induced expression of *LIM homeobox 8* (*Lhx8*), which is a key regulator of cholinergic neural function. The HSN2-OSR1/GSK3β-LHX8 pathway is therefore important for neurite outgrowth. Consistently, HSN2 mutants reported in HSANII patients suppressed SPAK and OSR1 activation and LHX8 induction. Interestingly, HSN2 mutants also suppressed neurite outgrowth by preventing interaction of between wild-type HSN2 and GSK3β. These results indicate that HSN2 mutants cause dysregulation of neurite outgrowth via GSK3β in the HSN2 and/or WNK1 pathways.

## Introduction

The with no lysine (WNK) family of serine/threonine protein kinases is conserved in many species^1,2^. There are four mammalian WNK family members, WNK1–4, and mutations in *WNK1* and *WNK4* cause a hereditary form of hypertension, pseudohypoaldosteronism type II (PHAII)^3^. WNK1 and WNK4 phosphorylate and activate SPAK and OSR1 kinases, which subsequently induces activation of ion co-transporters, such as NKCC1, NKCC2 and NCC in the kidney^4–7^. Moreover, *Wnk4*^*D561A*^ knockin mice exhibit phenotypes similar to PHAII^8^. These data indicate that dysregulation of the WNK-SPAK/OSR1 pathway causes unbalanced ion homeostasis and results in hypertension with hyperkalaemia in PHAII patients. The WNK1 has auto-inhibitory domain and this domain inhibits WNK1 auto-phosphorylation and phosphorylation of its substrate^9^. Similarly, the WNK4 also has auto-inhibitory domain and this domain suppresses WNK1 auto-phosphorylation and activation^10^. These findings suggest that the WNK kinases modulate the kinase activity of other WNK family members^11^. In fact, there is a report that WNK1 forms complexes with WNK1 and WNK4 and this interaction is important for the function of WNK1 activating downstream effectors^12^. Furthermore, it has been reported that WNK2 and WNK3 also bind to WNK1 and phosphorylate WNK1 inducing the activation of SPAK/OSR1^13^. These data suggest that the interaction between WNK family members have essential roles in WNK-SPAK/OSR1 pathway.

A neuron-specific *WNK1* splice variant, called HSN2, is expressed in the brain, dorsal root ganglia and sciatic nerve^14,15^. Mutations in a neural specific exon of *HSN2* cause the hereditary neuropathy, hereditary sensory and autonomic neuropathy type II (HSANII)^14,16–20^. HSANII is an autosomal recessive neuropathy characterized by sensory dysfunction, such as loss of pain, touch and temperature sensation^21^ and limb abnormalities, such as finger deformity and Charcot’s joint^20^. Knockdown of *HSN2* enhances K^+^-Cl^*−*^ cotransporter 2 (KCC2) expression and leads to abnormal neuromast development in zebrafish embryos^22^. Moreover, a knockout mouse lacking the *Hsn2* exon of *Wnk1* showed reduced pain hypersensitivity and restored γ-aminobutyric acid (GABA)-mediated depolarization after nerve injury^23^. Although these reports indicate that HSN2 has an essential neural function, the precise mechanism by which HSN2 mutants cause HSANII is still unclear.

LIM homeobox 8 (LHX8) is a LIM homeobox transcription factor and a key regulator of cholinergic neural function^24^. LHX8 is also involved in the determination of cholinergic or GABAergic cell fate^25^. Furthermore, LHX8 is required for cholinergic neurons in the ventral forebrain^26,27^. Consistent with these observations, we have reported that WNK1 and WNK4 induce LHX8 through OSR1 and GSK3β and that this signalling pathway is important for neurite outgrowth^28,29^. These findings indicate that not only WNK1 but also HSN2 regulates neurite outgrowth via LHX8 induced by OSR1 and GSK3β.

In this study, we analysed HSN2 functions in the OSR1-LHX8 pathway. We found that HSN2 was involved in the phosphorylation of SPAK and OSR1 and induced *Lhx8* expression to modulate neurite outgrowth identically to WNK1. We also demonstrated that HSN2 mutants suppressed OSR1 activation, *Lhx8* expression and effects of GSK3β on neurite outgrowth. These results reveal a novel role of HSN2 in neurite outgrowth and help to elucidate the pathogenic mechanism of HSANII.

## Results

### Wild-type but not mutant HSN2 binds to WNK1, WNK4, SPAK, OSR1 and HSN2

**Figure 1:**
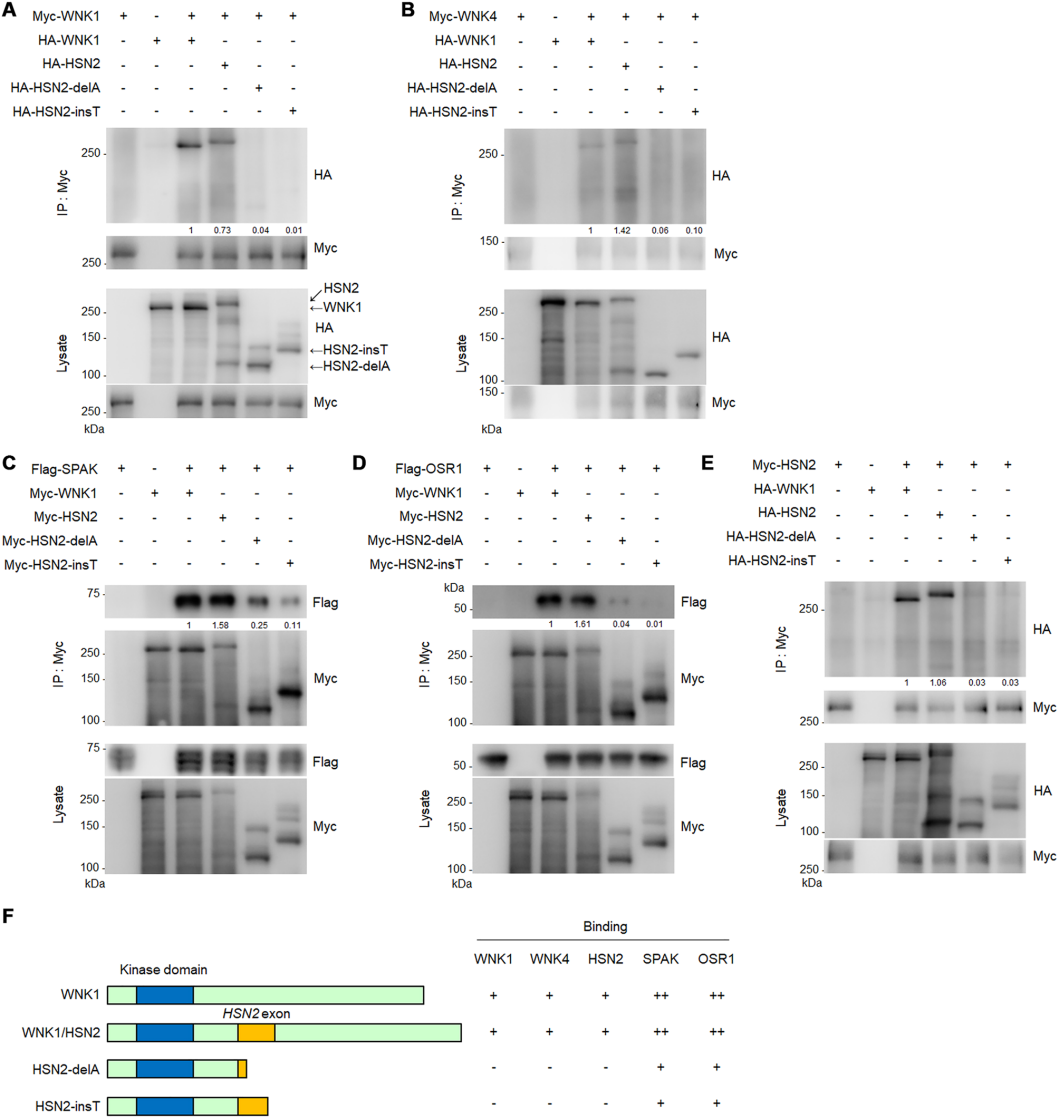
HSN2, but not HSN2 mutants, binds to WNK1, WNK4, SPAK, OSR1 and HSN2. (**A**–**E**) Interaction between WNK1 (**A**), WNK4 (**B**), SPAK (**C**), OSR1 (**D**) or HSN2 (**E**) and WNK1, HSN2, HSN2-delA and HSN2-insT was analysed in HEK293T cells by immunoprecipitation assays. Cells were transiently transfected with indicated vectors and 48 h later lysates were harvested and immunoprecipitated with an anti-Myc antibody for 4 h. Immunoprecipitates were subjected to immunoblotting with the indicated antibodies. (**F**) The schematic diagram of WNK1, HSN2 and its mutants was shown in left. The information of interaction levels of indicated proteins was shown in right.

*HSN2* is a neural specific isoform of *WNK1*, and mutations in *HSN2* cause the autosomal recessive neuropathy, HSANII^14^. WNK1 can form complexes with WNK1 and WNK4 to activate downstream effectors^12^. To analyse whether HSN2 binds to WNK1 and WNK4, we transiently expressed HA-tagged WNK1 and HSN2 with Myc-tagged WNK1 or WNK4 in HEK293T cells. Cell extracts were immunoprecipitated with a Myc antibody and immunoprecipitates were subjected to immunoblotting. As shown in Fig. 1A,B, HSN2 bound similarly to WNK1 and WNK4. In HSANII patients, mutations have been identified in the neural-specific alternatively spliced exon of *HSN2*, including a 1-bp deletion (2743delA^14^, referred to as HSN2-delA) and a 1-bp insertion (3237_3238insT^19^, referred to as HSN2-insT). These mutations cause a frameshift and result in truncated HSN2 proteins at amino acid 916 and 1080, respectively. We also checked the binding of these HSN2 mutants to WNK1 and WNK4 and found that they could not interact with WNK1 or WNK4 (Fig. 1A,B). WNK1 interacts with and phosphorylates the STE20 kinases, SPAK and OSR1, to activate the downstream pathway^5,6^; therefore, we analysed the binding of HSN2 and its mutants to SPAK and OSR1. We found that HSN2 and WNK1 bound similarly to SPAK and OSR1, but the binding of HSN2 mutants to SPAK and OSR1 was weak compared with that of WNK1 and HSN2 (Fig. 1C,D). Interestingly, HSN2 also bound to itself, but HSN2-delA and HSN2-insT could not bind to wild-type HSN2 (Fig. 1E). These results indicate that HSN2-delA and HSN2-insT mutants are unable to promote activation of downstream signalling because the HSN2 mutants could not interact with HSN2, WNK1, WNK4 or SPAK/OSR1 (Fig. 1F).

### HSN2 mutants, HSN2-delA and HSN2-insT, exhibit loss of neurite outgrowth function

**Figure 2:**
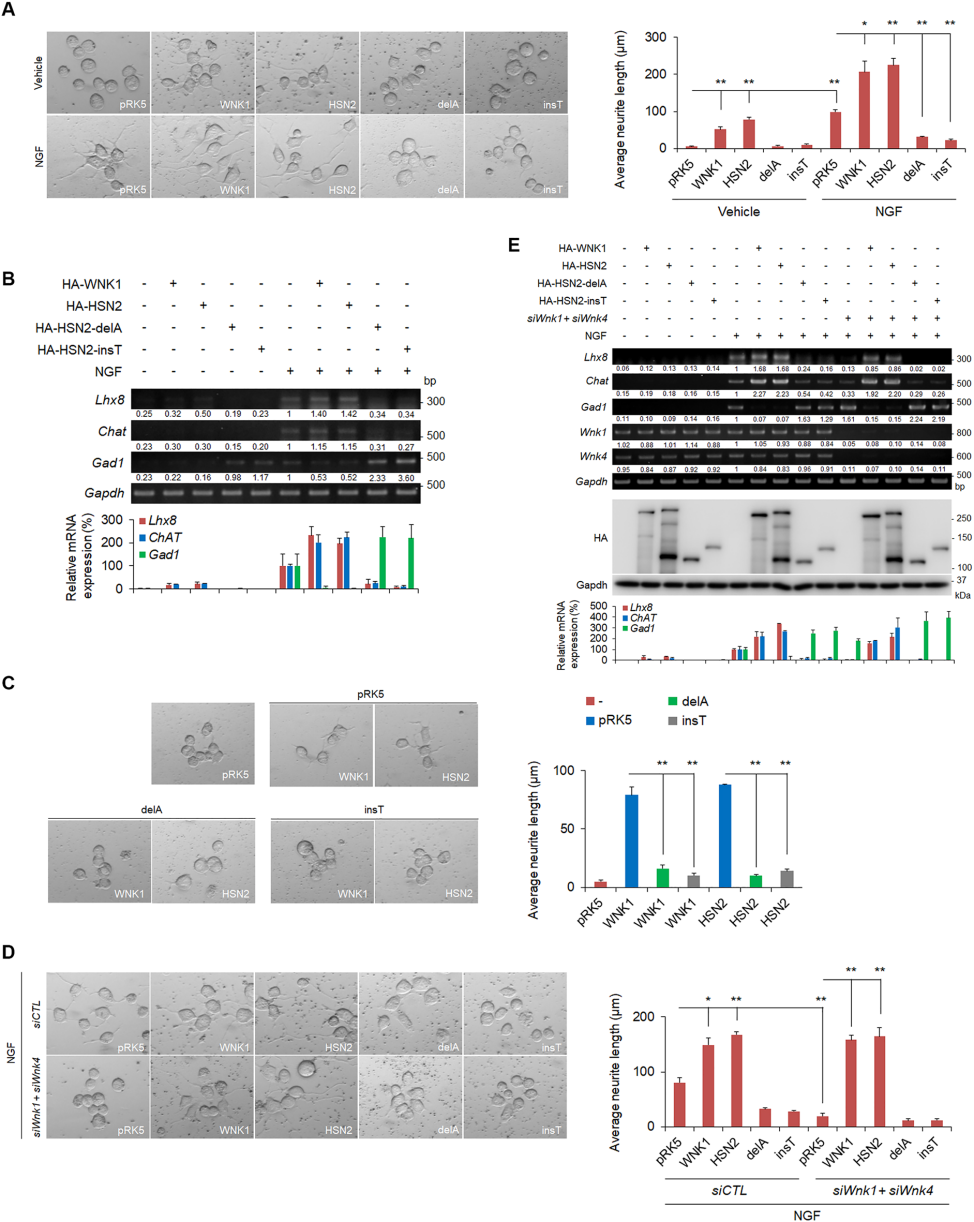
HSN2 mutants, HSN2-delA and HSN2-insT, exhibit loss of neurite outgrowth function. (**A**) Neurite outgrowth from Neuro2A cells treated with NGF (100 ng/ml) or vehicle-containing FBS-free medium for 24 h. Cells were transiently transfected with pRK5, HA-WNK1, HA-HSN2, HA-HSN2-delA or HA-HSN2-insT vectors 24 h prior to treatment. (**B**) RT-PCR and qPCR analysis of mRNA levels of neural marker genes, *Lhx8*, *ChAT* and *Gad1*, in Neuro2A cells indicated in (**A**). The value of each gene in empty vector-transfected, vehicle-treated cells was set to 1 and the value in empty vector-transfected, NGF-treated cells was set to 100. (**C**) Neurite elongation of Neuro2A cells 24 h after transfection of HA-WNK1 or HA-HSN2 with pRK5, HA-HSN2-delA or HA-HSN2-insT vectors. (**D**) Neurite outgrowth of Neuro2A cells treated with 100 ng/ml NGF-containing FBS-free medium for 24 h. Cells were transiently transfected with control siRNA (siCTL) or *siWnk1* and *siWnk4* 24 h prior to treatment. (**E**) RT-PCR and qPCR analysis of mRNA levels of *Lhx8*, *ChAT* and *Gad1* in Neuro2A cells indicated in (**C**). The value of each gene from vehicle-treated cells under siCTL treatment and from NGF-treated cells under siCTL treatment was set to 1 and 100, respectively.

We have previously reported that WNK1 and WNK4 are involved in induction of neural marker genes and neurite elongation in mouse neuroblastoma Neuro2A cells^28,29^. HSN2 may, therefore, have similar functions in neurite outgrowth and HSN2 mutants may exhibit loss of function. To examine whether HSN2 is involved in neurite outgrowth, we transiently expressed HSN2 in Neuro2A cells and observed that HSN2 induced neurite elongation identically to WNK1 (Fig. 2A upper panel). We then examined the effect of HSN2 mutants on neurite outgrowth. As shown in Fig. 2A, HSN2-delA and HSN2-insT mutants could not induce neurite elongation. Next, we checked the effects of wild-type and mutant HSN2 on neurite outgrowth induced by nerve growth factor (NGF). HSN2 constructs were transfected into Neuro2A cells and 24 h later cells were treated with serum-free medium containing 100 ng/ml NGF for 24 h. This treatment induced neurite elongation, and the expression of WNK1 or HSN2 enhanced this induction (Fig. 2A lower panel). In contrast, the expression of HSN2-delA or HSN2-insT suppressed neurite elongation in response to NGF-containing medium (Fig. 2A lower panel). We also analysed the expression of neural marker genes, *Lhx8* and *Choline acetyltransferase* (*ChAT*) for cholinergic neurons and *Glutamine acid decarboxylase 1* (*Gad1*) for GABAergic neurons. qPCR analysis indicated that NGF treatment greatly induced the expression of *Lhx8, ChAT* and *Gad1*, and that exogenous expression of WNK1 or HSN2 induced *Lhx8* and *ChAT* expression (Fig. 2B). WNK1 and HSN2 enhanced NGF-mediated expression of *Lhx8* and *ChAT*, but reduced that of *Gad1*. In contrast, expression of HSN2-delA and HSN2-insT mutants decreased *Lhx8* and *ChAT* expression and increased *Gad1* expression (Fig. 2B). These results indicate that WNK1 and HSN2 are involved in neurite outgrowth, but that HSN2-delA and HSN2-insT mutants fail in this activity. Interestingly, HSN2 mutants inhibited NGF-independent WNK1- and HSN2-mediated neurite elongation (Fig. 2C), suggesting that these mutants have a dominant negative effect on wild-type WNK1 and/or HSN2.

We next analyzed the effect of knockdown of *Wnk1* and *Wnk4* on neurite elongation of Neuro2A cells. We confirmed that treatment of *siWnk1* and *siWnk4* downregulated the expression of *Wnk1* and *Wnk4* in Neuro2A cells, respectively (Supplementary Fig. 1A). Since *Hsn2* gene includes the target sequence of *siWnk1*, *siWnk1* treatment has also reduced the expression of *Hsn2* (Supplementary Fig. 1A). While neurite elongation was suppressed by treatment of only *siWnk1,* but not *siWnk4*, the knockdown of both *Wnk1* and *Wnk4* more inhibited neurite elongation of Neuro2A cells (Supplementary Fig. 1B). To clarify this issue, we checked the endogenous expression levels of these genes in Neuro2A cells, and found that *Wnk4* and *Hsn2* mRNA were expressed at very low levels compared with *Wnk1* expression (Supplementary Fig. 1C). Especially, *Wnk4* expression level was less than one-thousandth of *Wnk1*. These results suggest that the knockdown of only *Wnk4* has no effect on neurite elongation, because *Wnk4* expression is very weak in Neuro2A cells. In addition, Wnk4 might have some functions for enhancing Wnk1 functions in neurite elongation of Neuro2A. We therefore used the both of *siWnk1* and *siWnk4*, because the knockdown of both *Wnk1* and *Wnk4* was the most effective for inhibiting neurite elongation. Moreover, we also analyzed whether Wnk1 and Wnk4 have redundancy in function of neurite elongation and found that WNK4 rescued the suppression of neurite elongation and neural maker genes caused by knockdown of only *Wnk1* and both *Wnk1* and *Wnk4* in Neuro2A cells (Supplementary Fig. 1D,E). This suggests that Wnk4 has functional redundancy with Wnk1 in neurite outgrowth of Neuro2A cells.

As shown in Fig. 2D, *siWnk1* and *siWnk4* treatment suppressed NGF-containing serum-free medium-mediated neurite elongation in Neuro2A cells. Moreover, knockdown of *Wnk1* and *Wnk4* suppressed *Lhx8* and *ChAT* expression, and induced *Gad1* expression (Fig. 2E), indicating that WNK proteins are required for neurite outgrowth of Neuro2A cells, as previously reported^28,29^. We next examined whether expression of HSN2 constructs could rescue these effects. We found that expression of WNK1 or HSN2 rescued neurite elongation and expression of neural marker genes (Fig. 2D,E). In contrast, these rescue effects were not observed with HSN2-delA or HSN2-insT mutants (Fig. 2D,E). These data indicate that HSN2 mutants fail to exert the normal functions of WNK1/HSN2 in neurite outgrowth.

### NGF activates SPAK/OSR1 through WNK1 and HSN2

**Figure 3:**
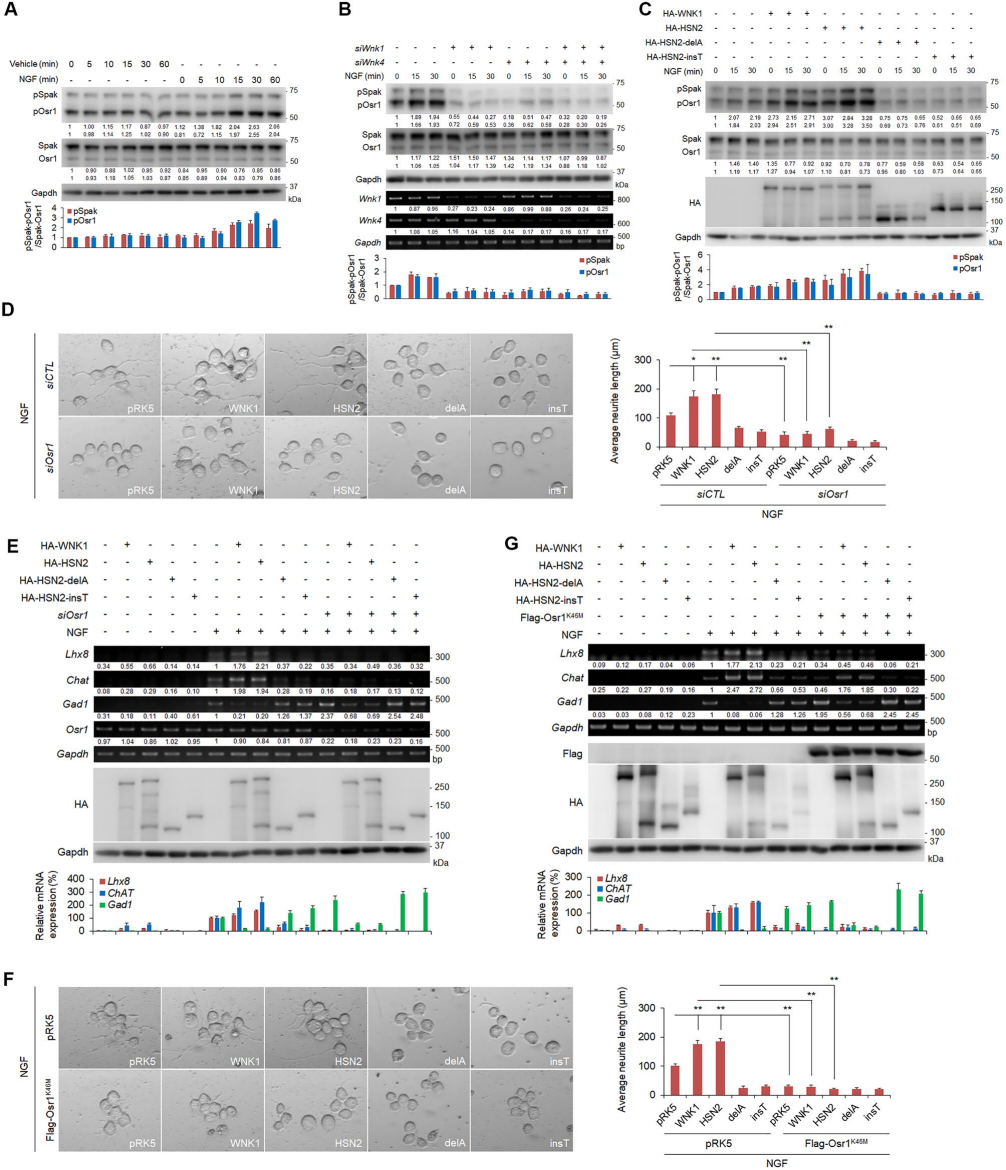
NGF activates SPAK/OSR1 through WNK1 and HSN2. (**A**) Immunoblot analysis of phosphorylated SPAK and OSR1 in Neuro2A cells treated with NGF (100 ng/ml) or vehicle-containing FBS-free medium for 0–60 min. (**B**) Phosphorylation of SPAK and OSR1 in Neuro2A cells treated with 100 ng/ml NGF-containing FBS-free medium for the indicated times. Cells were transfected with control siRNA (siCTL) or *siWnk1* and *siWnk4* 24 h prior to the treatment. (**C**) Phosphorylation of SPAK and OSR1 in Neuro2A cells treated with 100 ng/ml NGF-containing FBS-free medium for the indicated times. Cells were transiently transfected with pRK5, HA-WNK1, HA-HSN2, HA-HSN2-delA or HA-HSN2-insT vectors 24 h prior to the treatment. (**D**) Neurite outgrowth of Neuro2A cells treated with 100 ng/ml NGF-containing FBS-free medium for 24 h. Cells were transiently transfected with siCTL or *siOsr1* and indicated vectors 24 h prior to the treatment. (**E**) RT-PCR and qPCR analysis of mRNA levels of *Lhx8*, *ChAT* and *Gad1* in Neuro2A cells indicated in (**D**). The value of each gene from vehicle-treated cells under siCTL treatment and from NGF-treated cells under siCTL treatment was set to 1 and 100, respectively. (**F**) Neurite outgrowth of Neuro2A cells treated with 100 ng/ml NGF-containing FBS-free medium for 24 h. Cells were transiently transfected with empty vector or kinase-negative OSR1 (OSR1^K46M^) 24 h prior to treatment. (**G**) RT-PCR and qPCR analysis of mRNA levels of *Lhx8*, *ChAT* and *Gad1* in Neuro2A cells indicated in (**F**). The value of each gene from empty vector-transfected, vehicle-treated cells and from empty vector-transfected, NGF-treated cells was set to 1 and 100, respectively. The band intensities of pSpak and pOsr1 in (**A**–**C**) by normalising intensities to the Spak and Osr1 signal, respectively. The measured values were shown in the graph under each immunoblot data.

To analyse whether HSN2 regulates activation of SPAK and OSR1 in neurite outgrowth, we checked phosphorylation of SPAK and OSR1 in Neuro2A cells treated with NGF-containing serum-free medium. The phosphorylation of SPAK and OSR1 was observed at 10 to 15 min after treatment with NGF-containing medium (Fig. 3A). In contrast, knockdown of either *Wnk1* or *Wnk4* suppressed the phosphorylation of SPAK and OSR1 by NGF (Fig. 3B). Moreover, knockdown of both *Wnk1* and *Wnk4* strongly inhibited SPAK and OSR1 phosphorylation (Fig. 3B). We also checked the phosphorylation of CATCHtide peptide by OSR1 with treatment of NGF, because the CATCHtide was reported as a peptide substrate of SPAK and OSR1^30^. Analysis of SDS-PAGE including Phos-tag showed that OSR1 from NGF-treated Neuro2A cells induced an electrophoretic mobility shift of CATCHtide indicating the phosphorylation of CATCHtide (Supplementary Fig. 2). These findings demonstrate that NGF activates the WNK-SPAK/OSR1 pathway in Neuro2A cells. We next analysed the effect of HSN2 constructs on the phosphorylation of SPAK and OSR1 mediated by NGF. We found that HSN2 expression increased the level of SPAK and OSR1 phosphorylation identically to that following WNK1 expression (Fig. 3C). By contrast, the expression of HSN2-delA and HSN2-insT mutants suppressed the NGF-induced phosphorylation of SPAK and OSR1 (Fig. 3C). These results indicate that HSN2 mutants cannot activate SPAK and OSR1 and work in a dominant negative manner. These data are consistent with the neurite elongation and neural marker gene expression results (Fig. 1A–C).

We previously demonstrated that the WNK1-OSR1 pathway is involved in neurite outgrowth^28^. To confirm whether NGF-induced neurite outgrowth is mediated by the HSN2-OSR1 pathway, we examined the effect of *Osr1* knockdown on neurite outgrowth. As expected, WNK1 expression enhanced neurite outgrowth induced by NGF treatment, and knockdown of *Osr1* inhibited this elongation (Fig. 3D). Similar results were obtained with HSN2 expression and knockdown of *Osr1* (Fig. 3D). In contrast, expression of HSN2 mutants suppressed NGF-induced neurite elongation, and knockdown of *Osr1* completely inhibited neurite outgrowth (Fig. 3D). RT-PCR and qPCR analysis showed that NGF-induced *Lhx8* and *ChAT* expression was completely suppressed by *Osr1* knockdown even though WNK1 or HSN2 was expressed in Neuro2A cells (Fig. 3E). Moreover, although NGF-induced *Gad1* expression was reduced by WNK1 or HSN2 expression, knockdown of *Osr1* rescued this reduction (Fig. 3E). We also examined the effect of a kinase-negative form of OSR1 (OSR1^K46M^), and similar results were obtained to those with *Osr1* knockdown (Fig. 3F,G). These results demonstrate that NGF-induced neurite outgrowth via WNK1 and HSN2 require OSR1 activity.

### The HSN2-OSR1-LHX8 pathway is important for NGF-induced neurite outgrowth

**Figure 4:**
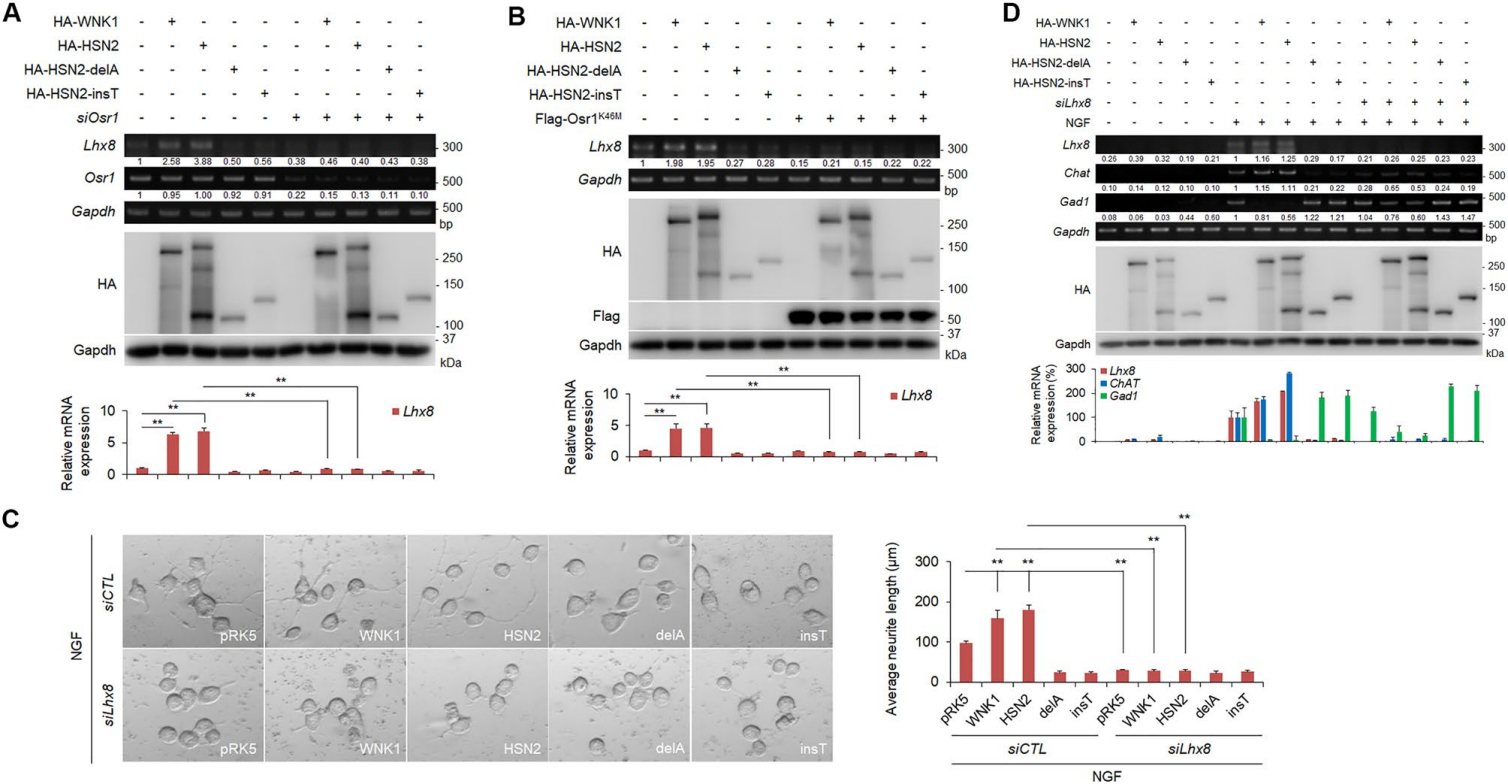
The HSN2-OSR1-LHX8 pathway is important for NGF-induced neurite outgrowth. (**A**,**B**) RT-PCR and qPCR analysis of *Lhx8* mRNA levels in Neuro2A cells 24 h after transfection with pRK5, HA-WNK1, HA-HSN2, HA-HSN2-delA or HA-HSN2-insT vectors and control siRNA (siCTL) or *siOsr1* (**A**) and Flag-OSR1^K46M^ (**B**). **P < 0.01. (**C**) Neurite outgrowth of Neuro2A cells treated with 100 ng/ml NGF-containing FBS-free medium for 24 h. Cells were transiently transfected with siCTL or *siLhx8* and indicated vectors 24 h prior to treatment. (**D**) RT-PCR and qPCR analysis of mRNA levels of *Lhx8*, *ChAT* and *Gad1* in Neuro2A cells indicated in (**C**). The value of each gene from vehicle-treated cells under siCTL treatment and from NGF-treated cells under siCTL treatment was set to 1 and 100, respectively.

LHX8 is a key regulator of cholinergic neural function and is involved in the determination of cholinergic and GABAergic cell fate^24,25^. We previously demonstrated that the WNK1-OSR1 pathway is important for *Lhx8* expression^28^. To determine whether *Lhx8* expression is mediated by the HSN2-OSR1 pathway, we examined the effects of HSN2 constructs on Neuro2A cells in the absence of NGF stimulation. We confirmed that expression of WNK1 or HSN2 mediated *Lhx8* induction, and that knockdown of *Osr1* suppressed this induction (Fig. 4A). We also examined the effect of a kinase-negative OSR1^K46M^ mutant on *Lhx8* expression, and found that OSR1^K46M^ also suppressed *Lhx8* expression induced by WNK1 or HSN2 expression (Fig. 4B). In contrast, HSN2-delA and HSN2-insT did not induce *Lhx8* expression (Fig. 4A,B). We next analysed whether HSN2-induced neurite outgrowth in Neuro2A cells is mediated by LHX8. *Lhx8* knockdown inhibited not only WNK1- but also HSN2-induced neurite outgrowth (Fig. 4C). Furthermore, although the expression of HSN2 caused an increase in *ChAT* expression and a decrease in *Gad1* expression, knockdown of *Lhx8* suppressed *ChAT* expression and enhanced *Gad1* expression by the expression of HSN2 (Fig. 4D). These results indicate that HSN2-induced neurite outgrowth, including neurite elongation and expression of cholinergic neuron marker genes, was mediated by LHX8 through OSR1 activity.

### HSN2 mutants suppress neurite outgrowth via GSK3β

**Figure 5:**
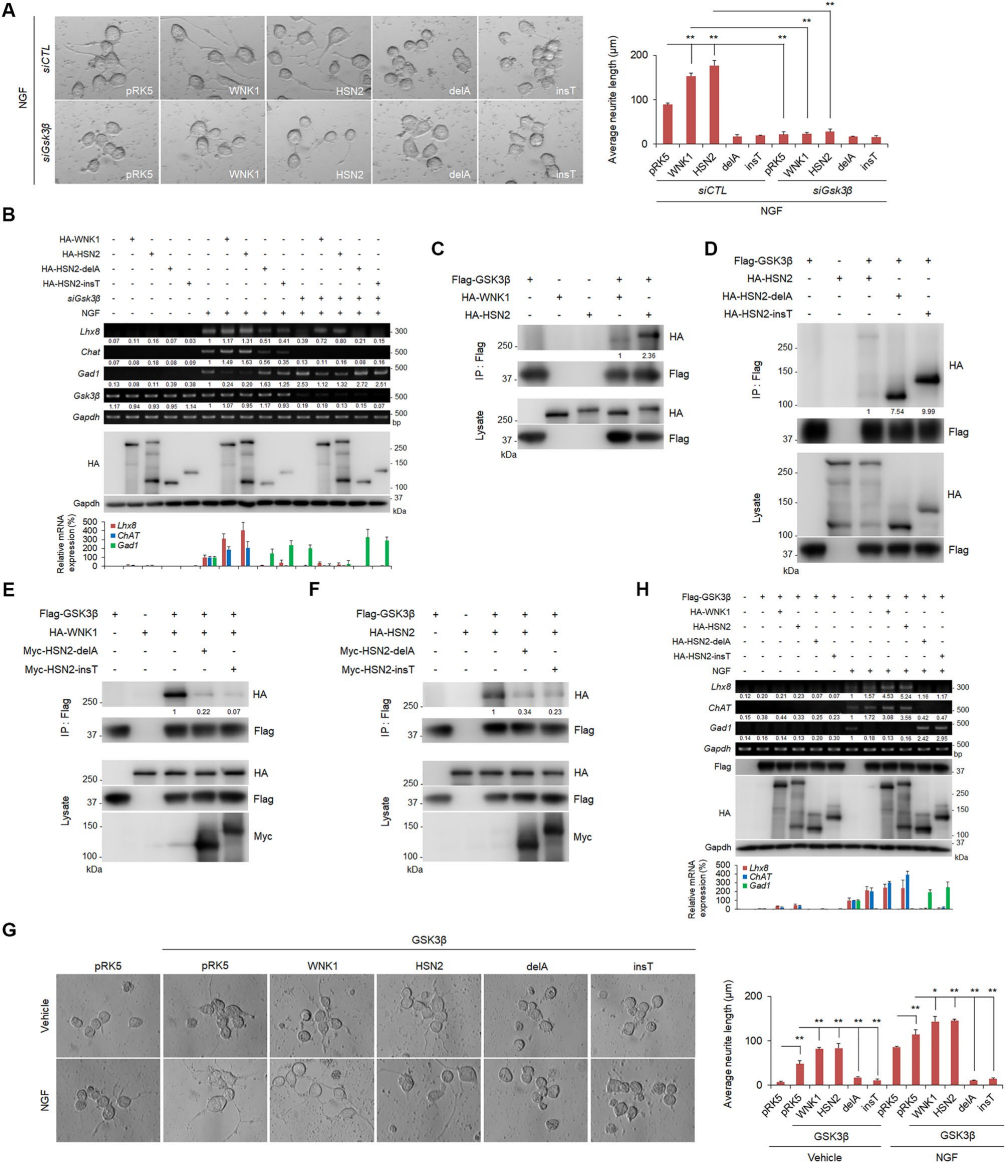
HSN2 mutants suppress GSK3β functions in neurite outgrowth. (**A**) Neurite outgrowth of Neuro2A cells treated with 100 ng/ml NGF-containing FBS-free medium for 24 h. Cells were transiently transfected with control siRNA (siCTL) or *siGsk3β* and indicated vectors 24 h prior to treatment. (**B**) RT-PCR and qPCR analysis of mRNA levels of *Lhx8*, *ChAT* and *Gad1* in Neuro2A cells indicated in (**A**). The value of each gene from vehicle-treated cells under siCTL treatment and from NGF-treated cells under siCTL treatment was set to 1 and 100, respectively. (**C**,**D**) Binding analysis of WNK1 and HSN2 to GSK3β (**C**), and of HSN2-delA and HSN2-insT mutants to GSK3β (**D**). Cells were transiently transfected with indicated vectors and 48 h later lysates were immunoprecipitated with a Flag antibody for 4 h. Immunoprecipitates were subjected to immunoblotting assays with the indicated antibodies. (**E**,**F**) Interaction between WNK1 (**E**) or HSN2 (**F**) and GSK3β with and without HSN2-delA and HSN2-insT mutants was analysed in HEK293T cells by immunoprecipitation assays. Cells were transiently transfected with indicated vectors and 48 h later lysates were immunoprecipitated with a Flag antibody for 4 h. Immunoprecipitates were subjected to immunoblotting assays with the indicated antibodies. (**G**) Neurite elongation of Neuro2A cells treated with 100 ng/ml NGF-containing FBS-free medium for 24 h. Cells were transiently transfected with the indicated vectors 24 h prior to treatment. (**H**) RT-PCR and qPCR analysis of mRNA levels of *Lhx8*, *ChAT* and *Gad1* in Neuro2A cells indicated in (**G**). The value of each gene from control vector-transfected and vehicle- or NGF-treated cells was set to 1 and 100, respectively.

GSK3β is a positive effector of WNK signalling in neurite outgrowth^29^. To confirm whether HSN2 mediates NGF-induced neurite outgrowth through GSK3β, we examined the effect of *Gsk3β* knockdown on neurite outgrowth of Neruo2A cells. As shown in Fig. 5A, WNK1- and HSN2-mediated neurite elongation was suppressed by *siGsk3β* treatment. Moreover, HSN2 mutants suppressed NGF-induced neurite elongation, and knockdown of *Gsk3β* completely inhibited neurite outgrowth (Fig. 5A). We also analysed expression of neural marker genes in Neuro2A cells and found that *Gsk3β* knockdown suppressed NGF-induced *Lhx8* and *ChAT* expression, even though WNK1 or HSN2 was expressed (Fig. 5B). Furthermore, although WNK1 or HSN2 expression decreased NGF-induced *Gad1* expression, knockdown of *Gsk3β* rescued this reduction (Fig. 5B). These results demonstrate that NGF-induced neurite outgrowth via WNK1 and HSN2 requires not only OSR1 activity but also GSK3β.

We next analysed the interaction between GSK3β and HSN2, and found that HSN2 interacted more strongly with GSK3β than with WNK1 (Fig. 5C). Interestingly, HSN2 mutants associated more robustly with GSK3β than with wild-type HSN2 (Fig. 5D). From these results, we predicted that HSN2 mutants might suppress the binding of WNK1 and HSN2 to GSK3β by covering the binding sites of GSK3β. To confirm this hypothesis, we transiently expressed Flag-GSK3β and HA-WNK1 or HA-HSN2 with or without Myc-HSN2-delA and Myc-HSN2-insT mutants in HEK293T cells and then immunoprecipitated cell extracts with a Flag antibody. The binding of WNK1 and HSN2 to GSK3β was inhibited by expression of each HSN2 mutant (Fig. 5E,F). Moreover, these mutants also prevented the homo-dimerization of WNK1 and HSN2 respectively (Supplementary Fig. 3A,B), indicating that HSN2 mutants suppress the function of GSK3β in the WNK1/HSN2 pathway by preventing the interaction.

We then examined the effect of HSN2 mutants on GSK3β function in neurite outgrowth. As shown in Fig. 5G, GSK3β expression weakly induced neurite elongation of Neuro2A cells and WNK1 and HSN2 enhanced this induction under treatment with vehicle or NGF. In contrast, HSN2 mutants inhibited the GSK3β-induced neurite elongation (Fig. 5G). Consistent with these results, RT-PCR and qPCR analysis showed that GSK3β elevated NGF-induced *Lhx8* and *ChAT* expression and decreased *Gad1* expression (Fig. 5H). In addition, although wild-type HSN2 enhanced the expression of *Lhx8* and *ChAT*, HSN2 mutants suppressed the induction of these genes and facilitated *Gad1* expression (Fig. 5H). These results indicate that HSN2 mutants suppress the neurite outgrowth function of GSK3β by preventing the interaction of WNK1 and HSN2 with GSK3β.

### The function of HSN2 mutants and GSK3β in mouse primary neuron

**Figure 6:**
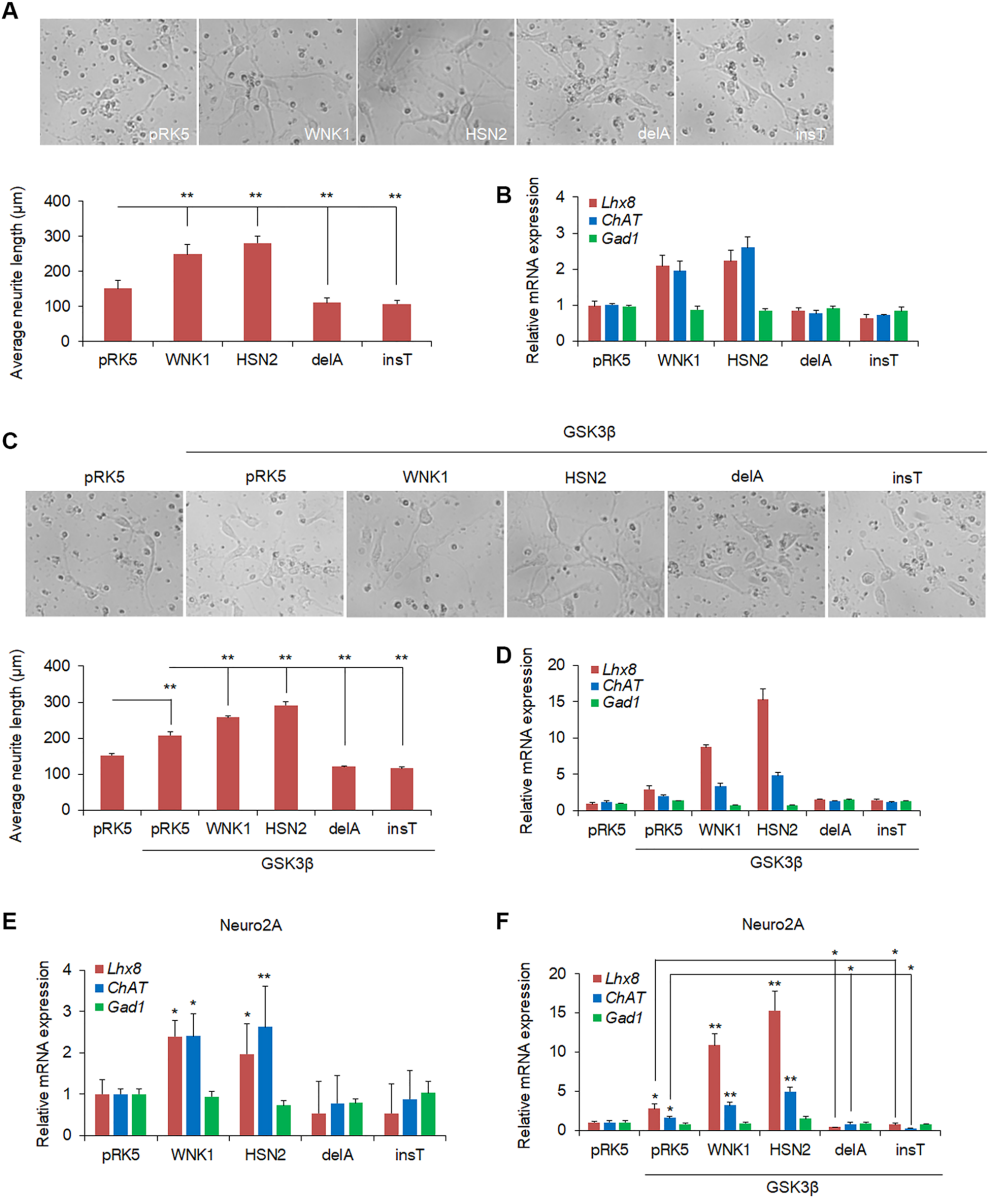
The function of HSN2 mutants and GSK3β in mouse primary neuron. (**A**) Neurite outgrowth of mouse primary cortical neural cells expressing indicated plasmids. Average neurite length was quantified by ImageJ software with NeuronJ plugin. (**B**) qPCR analysis of mRNA levels of *Lhx8*, *ChAT* and *Gad1* in primary neural cells indicated in (**A**). (**C**) Neurite elongation and average neurite length of mouse primary cortical neural cells expressing indicated plasmids. (**D**) The expression levels of *Lhx8*, *ChAT* and *Gad1* mRNA in primary neural cells indicated in (**C**). (**E**,**F**) The expression levels of *Lhx8*, *ChAT* and *Gad1* mRNA in non-treated Neuro2A cells expressing indicated plasmids. *P < 0.05, **P < 0.01.

Finally, we analyzed the effect of WNK1, HSN2 and HSN2 mutants on neurite outgrowth of mouse primary cortical neuron. Consistent with the results from Neuro2A, neurite elongation of primary neuron was enhanced by WNK1 and HSN2 expression, but suppressed by HSN2 mutants (Fig. 6A). The qPCR analysis showed that *Lhx8* and *ChAT* expression were induced by WNK1 and HSN2 expression (Fig. 6B). However, *Gad1* expression was not changed by WNK1, HSN2 and HSN2 mutants, and the repressive effect of HSN2 mutants on *Lhx8* and *ChAT* expression was not confirmed (Fig. 6B). We also examined whether HSN2 mutants have dominant negative function for GSK3β in primary neural cells. The result showed that GSK3β expression induced the elongation of neurite in primary neural cells and WNK1 and HSN2 enhanced GSK3β-mediated neurite elongation, but HSN2 mutants repressed the function of GSK3β (Fig. 6C). Moreover, GSK3β induced the expression of *Lhx8* and *ChAT,* but not *Gad1*, and WNK1 and HSN2 facilitated GSK3β-mediated inductions, but HSN2 mutants suppressed these events in primary neuron (Fig. 6D). We also analyzed whether similar results were obtained in non-treated Neuro2A cells, and found that WNK1 and HSN2 increased *Lhx8* and *ChAT* expression, but HSN2 mutants had no effect on these genes expression, and *Gad1* was not regulated by WNK1/HSN2 constructs in basal state of Neuro2A cells (Fig. 6E). In contrast, GSK3β-mediated induction of *Lhx8* and *ChAT* was repressed by HSN2 mutants (Fig. 6F). These results indicate that WNK1, HSN2 and HSN2 mutants have similar effect in primary neural cells and Neuro2A cells.

## Discussion

HSANII is a hereditary neuropathy that is characterized by sensory dysfunction, including loss of pain, touch and temperature sensation^21^. HSANII is caused by mutations in a neuron specific exon of *HSN2*, which is a neural-specific splice variant of the *WNK1* gene. These mutations include HSN2-delA and HSN2-insT^14,19^. Although HSN2 is predicted to have an essential function in neurons, the precise mechanism by which HSN2 mutants cause HSANII is still unclear. In this study, we made constructs of HSN2, including HSN2-delA and HSN2-insT mutants, and analysed their effects on the WNK/HSN2 pathway and neurite outgrowth using Neuro2A cells. WNK1 forms complexes with WNK1 and WNK4 to promote activation of itself and to activate the downstream effectors, SPAK and OSR1^28,29^. We showed that HSN2 bound to WNK1 and WNK4 (Fig. 1A,B), and formed a complex with itself (Fig. 1E). These results indicate that HSN2 is also activated by forming a homodimer and auto-phosphorylation. We also observed that NGF induced SPAK and OSR1 phosphorylation and that the phosphorylation levels were enhanced by HSN2 expression (Fig. 3C), indicating that HSN2 activates the SPAK/OSR1 pathway identically to WNK1. We previously reported the involvement of the WNK1-OSR1 pathway in neurite outgrowth^28^. Therefore, we postulated that the HSN2-OSR1 pathway is also involved in regulating neurite outgrowth. As expected, knockdown of *Osr1* suppressed HSN2-mediated neural elongation and expression of the cholinergic neural markers, *Lhx8* and *ChAT* (Fig. 3D,E). In addition, kinase-negative OSR1^K46M^ expression also inhibited HSN2-mediated neurite elongation and *Lhx8* and *ChAT* expression (Fig. 3F,G). These results indicate that activation of OSR1 mediated by HSN2 is required for neurite outgrowth. In contrast to HSN2, HSN2-delA and HSN2-insT mutants did not interact with WNK1, WNK4 or HSN2, and weakly bound to SPAK and OSR1 compared with wild-type HSN2 (Fig. 1). Additionally, we showed that expression of HSN2-delA and HSN2-insT mutants suppressed NGF-induced phosphorylation of SPAK and OSR1 (Fig. 3C). These data indicate that HSN2 mutants are not able to activate the SPAK/OSR1 pathway even though they include the kinase domain. One study has reported that the C-terminal domain of WNK isoforms is important for interaction and auto-phosphorylation of WNK kinases^13^, suggesting that the C-terminal domain of the WNK1 isoform, HSN2, is required for its self-activation. Most mutations in *HSN2* occur in the neural-specific *HSN2* exon and cause deletion of subsequent domains, for example, HSN2-delA and HSN2-insT^19^. We therefore consider that the C-terminal domain of HSN2 is required for the activation of SPAK and OSR1 and that the HSN2 mutants lacking C-terminal domain inhibit this activation and might have some functions in the pathological mechanism of HSANII.

We showed that HSN2 regulates neurite outgrowth through *Lhx8* induction in Neuro2A cells (Fig. 4D). In addition, OSR1 activation was required for HSN2-mediated *Lhx8* expression (Fig. 4A,B). These results indicate that the HSN2-OSR1-LHX8 pathway is important for neurite outgrowth. In contrast, HSN2-delA and HSN2-insT mutants, which are observed in HSANII patients, could not induce activation of SPAK and OSR1, or expression of *Lhx8* (Figs. 3C and 4A,B), indicating that HSANII might be caused by dysregulation of neurite outgrowth via HSN2 mutant-mediated suppression of the HSN2-OSR1-LHX8 pathway. However, concerning the pathological mechanism of HSANII, knockdown of *HSN2* in the zebrafish embryo enhances expression of ion co-transporter, KCC2, and leads to abnormal neuromast development^22^. Moreover, *HSN2*-deficient mice display increased KCC2 activity and decreased levels of Cl^−^ in nerves^23^. These reports suggest that the regulation of KCC2 and ion-homeostasis by HSN2 is important for normal neural development. Here, we showed that the SPAK/OSR1 pathway was similarly activated by HSN2 and WNK1. Because WNK1-mediated activation of SPAK/OSR1 regulates the concentration of Cl^*−*^ ions in neurons through KCC2^31^, the HSN2-SPAK/OSR1 pathway might regulate not only *Lhx8* expression but also ion concentrations in neurons. Therefore, *HSN2* mutation can dysregulate neurite outgrowth and ion-concentrations in neurons, and might cause loss of pain, touch and temperature sensation in HSANII patients.

Previous reports provide strong evidence that GSK3β has essential roles in neurodevelopmental processes^32,33^. We have also reported GSK3β to be a positive downstream regulator in the WNK-SPAK/OSR1 pathway, which regulates neurite outgrowth^29^. Here, we found that GSK3β also has a positive effect on the HSN2 pathway in Neuro2A cells and mouse primary cortical neurons (Figs. 5A,B, 6C). In contrast, HSN2-delA and HSN2-insT mutants suppressed GSK3β-mediated neurite elongation and specification (Figs. 5G,H, 6C,D). Interestingly, HSN2 mutants interacted more strongly with GSK3β than with wild-type HSN2 and prevented the binding of WNK1 and HSN2 to GSK3β (Fig. 5D–F). These results indicate that HSN2 mutants suppress GSK3β in neurite outgrowth by inhibiting its binding to WNK1 and HSN2. Conditional knockout mice of both GSK3α and GSK3β isoforms in neural progenitors has shown that GSK3s are essential for cortical neurogenesis^34^; however, GSK3α-S21A/GSK3β-S29A double knock-in mice show that phosphorylation of GSK3s at their N-terminal serine residues is not necessary for neural polarization, including neurite outgrowth^35^. In support of these findings, we reported that GSK3β is a downstream effector of the WNK-SPAK/OSR1 pathway in the regulation of neurite outgrowth, but neither WNK1 nor OSR1 kinases can phosphorylate GSK3β^29^. Therefore, formation of GSK3β, WNK1/HSN2 and/or SPAK/OSR1 complexes has essential roles in neurite outgrowth and HSN2 mutants might cause HSANII by inhibiting formation of these complexes. However, further studies are needed to identify the precise mechanism of HSANII pathology.

In conclusion, our results demonstrate a critical role for HSN2 and suppressive effects of HSN2 mutants on the functions of GSK3β in neurite outgrowth. These findings may be beneficial for understanding the pathogenesis of HSANII.

## Materials and methods

### Cell culture and treatment

HEK293T and Neuro2A cells were cultured in DMEM (Gibco, Waltham, MA, USA) containing 10% foetal bovine serum (FBS). For neuronal differentiation, Neuro2A cells were treated with serum-free DMEM containing 100 ng/ml nerve growth factor (NGF) for 24 h. Images of neurite outgrowth from Neuro2A cells were obtained using Axioscope microscopes (Carl Zeiss, Germany), and the length of each neurites was quantified using ImageJ software with NeuronJ plugin^36^.

### Antibodies

Polyclonal antibodies, Anti-DDDDK, anti-Myc and anti-HA were obtained from Medical and Biological Laboratories (Japan). Monoclonal antibody, anti-GAPDH (5A12), was purchased from Wako (Japan). Anti-SPAK/OSR1 and anti-phospho-SPAK/OSR1 antibodies were prepared as previously described^6^. These antibodies were used for immunoblotting at 1/1000. Anti-Myc (9B11) monoclonal antibody was purchased from Cell Signaling Technology (Danvers, MA, USA) and anti-Flag (M2) monoclonal antibody was obtained from Sigma-Aldrich (St. Louis, MO, USA), and these antibodies were used for immunoprecipitation at 1/500.

### Transfection of expression vectors and siRNA

The coding sequence of the human *HSN2* gene (NM_001184985) was cloned into the pRK5 vector with different molecular tags (Myc and HA). *HSN2* mutants (2743delA^14^ and 3237_3238insT^19^) were also cloned into pRK5. The sequence of these constructs was confirmed using a 3130 Genetic Analyzer (Applied Biosystems, Waltham, MA, USA). Other plasmids used were: HA-WNK1, Myc-WNK1, HA-WNK4, Flag-SPAK, Flag-OSR1, Flag-mOsr1^K46M^ and Flag-GSK3β in pRK5. Cultured cells were transfected with these constructs using polyethyleneimine (Polysciences, Warrington, PA, USA). We also performed reverse transfection of siRNA using the TransIT-X2 Dynamic Delivery System (Mirus Bio, Madison, WI, USA). siRNA mouse target sequences were: *Wnk1* 5′-GAUAGGGUGUCCUUAAUUA-3′ (NM_001185020.1 2701–2719 bp), *Wnk4* 5′-CUACUCCGAGUGUCAGAAUGC-3′ (NM_175638.3 1218–1236 bp), *Osr1* 5′-GAUAUUCGAUUUGAAUUUA-3′ (NM_133985.2 1698–1716 bp), *Lhx8* 5′-GGAAGAAAUGGCUUAUUCU-3′ (NM_010713.2 1194–1212 bp) and *Gsk3β* 5′-GAAAUGAACCCAAAUUAUA-3′ (NM_019827.7 2372–2390 bp).

### Reverse-transcription polymerase chain reaction (RT-PCR) analysis

Total RNA was isolated using TRI Reagent (Molecular Research Center, Cincinnati, OH, USA). Double-strand cDNA was prepared from total RNA using oligonucleotide (dT), random primers and Moloney murine leukaemia virus reverse transcriptase (Invitrogen, Carlsbad, CA, USA). *Gapdh* was used to normalize the cDNA samples. The intensity of each band was quantified using ImageJ software. For quantification, the intensity of the *Gapdh* band was used to normalise each DNA signal. The mouse sequences of the primer pairs for PCR were as follows: *Wnk1* 5′-AGAGGATGGCTCAGGTAGTCCACAC-3′ and 5′-AACACACAGCTGCCCAGGAGCAGAG-3′, *Wnk4* 5′-AAGCTCTGGCTGCGCATGGAGGATG-3′ and 5′-GGATCGAGGTCTCCGTCGAAGAGTC-3′, *Osr1* 5′-TGGCCGTCTCCATAAGACAGAGGAC-3′ and 5′-TATCCGAGCCTTCAACACCAGATGC-3′, *Gsk3β* 5′-GCAGCAAGGTAACCACAGTAGTGGC-3′ and 5′-TGGTGCCCTGTAGTACCGAGAACAG-3′, *Lhx8* 5′-GACCCAGCTGCCAATAAGTCATACC-3′ and 5′-GACACACACTCGAGCCAACTATCTC-3′, *ChAT* 5′-CAGTGCATGCAACACCTGGTACCTG-3′ and 5′-GAACAGATCACCCTCACTGAGACGG-3′, *Gad1* 5′-CATCTTCCACTCCTTCGCCTGCAAC-3′ and 5′-CAGTCAACCAGGATCTGCTCCAGAG-3′, *Gapdh* 5′-GCCATCACTGCCACCCAGAAGACTG-3′ and 5′-CATGAGGTCCACCACCCTGTTGCTG-3′.

### Quantitative real-time PCR

Quantitative PCR was performed using a 7300 Real-Time PCR Cycler (Applied Biosystems) and THUNDERBIRD SYBR qPCR Mix (TOYOBO, Japan). The primer sequences were as follows: *Wnk1* 5′-AAGATGAAAGATATTCCATC-3′ and 5′-TTCTTAATATCTTCAATACG-3′, *Wnk4* 5′-TCGGGCACAAAGCCCAACAG-3′ and 5′-CTTATCCGTGCGGATGCAGC-3′, *Hsn2* 5′-GGAGAGTGTCCTGCCTATGC-3′ and 5′-CGGATGCGCCATGGACTGAG-3′. The sequences of the primer pairs for mouse *Lhx8*, *ChAT*, *Gad1* and *Gapdh* were described previously^37–39^. *Gapdh* was used to normalize the cDNA samples.

### Immunoprecipitation and immunoblot analysis

Cells were lysed with TNE buffer [10 mM Tris–HCl (pH 7.4), 0.1% NP-40, 150 mM NaCl, 1 mM EDTA, 1 mM DTT and protease inhibitor cocktail cOmplete (Roche, Switzerland)]. Lysates were pre-cleared with Protein A/G PLUS-agarose (Santa Cruz Biotechnology, Dallas, TX, USA) and immunoprecipitated with the indicated antibodies. For immunoblot analysis, cell lysates or immunoprecipitates were resolved by sodium dodecyl sulphate-polyacrylamide gel electrophoresis (SDS-PAGE) and then transferred to polyvinylidene difluoride membranes (Merck Millipore, Germany). The membranes were probed with primary antibodies, followed by incubation with horseradish peroxidase-conjugated mouse or rabbit immunoglobulin G (GE Healthcare) and visualized using Immobilon Western (Merck Millipore). The protein bands were digitalized using the image analyser LAS-4000 Mini (Fujifilm, Japan), and the intensity of each band was quantified using ImageJ software. For quantification, the intensity of the Gapdh band was used to normalise each protein signal.

### In vitro kinase assay

OSR1 substrate CATCHtide^30^ was constructed in pGEX 4T-1 plasmid and GST-tagged CATChtide was extracted from *E. coli* BL21 strain. Empty vector or Flag-mOsr1 was transfected into Neuro2A cells and cells were treated with vehicle, NGF (100 ng/ml) or sorbitol (500 mM) for indicated time. After stimulation, cells were lysed with TNE buffer and Flag-mOsr1 was immunoprecipitated with Flag-antibody and Protein A/G PLUS-agarose. Immunoprecipitates and GST-tagged CATCHtide were incubated in kinase buffer containing 10 mM HEPES (pH 7.4), 1 mM DTT, 5 mM MgCl_2_, and 50 μM ATP at 37 °C for 30 m. Phosphorylated CATCHtide was subjected to SDS-PAGE including Phos-tag (Fujifilm) and separated from non-phosphorylated CATCHtide in the gel. The intensity of each band was quantified using ImageJ software. For quantification, the intensity of the CBB-stained band was used to normalise each protein signal.

### Preparation and treatment of mouse primary cortical neuron

Mouse primary cortical neurons were obtained as previously reported^40^. The isolated cells were plated at 5 × 10^5^ cells in six well-plate with Neurobasal Medium (Gibco) containing B-27 Supplement (Gibco). Plate was pre-coated with Poly-d-Lysine (Sigma-Aldrich). After 3 days of plating, cells were transfected with indicated plasmids using TransIT-X2 Dynamic Delivery System. After 2 days of transfection, neurite elongation was observed by Axioscope microscopes, and the length of each neurites was quantified using ImageJ software with NeuronJ plugin. After the observation, mRNA was extracted in these cells for the quantitative PCR.

### Statistical analysis

All experiments were independently repeated at least three times. Data are presented as the mean ± standard deviation (SD). Statistical analysis was performed with Student’s *t* test using Microsoft Excel (Microsoft, DC, USA). P < 0.05 was considered statistically significant.

## Supplementary Information

Supplementary Figures.

## Data Availability

The data are available from the corresponding author upon request.
