# Supplementary material for: WNK1/HSN2 mediates neurite outgrowth and differentiation via a OSR1/GSK3β-LHX8 pathway
Source: Sci Rep. 2022 Sep 23;12:15858. doi: 10.1038/s41598-022-20271-y (PMC9508073; doi:10.1038/s41598-022-20271-y)

**Supplementary Information**

**Title**

WNK1/HSN2 mediates neurite outgrowth and differentiation via a OSR1/GSK3β-LHX8 pathway

**Authors**

Masahiro Shimizu, Hiroshi Shibuya,

**Supplementary Figures 1-4**


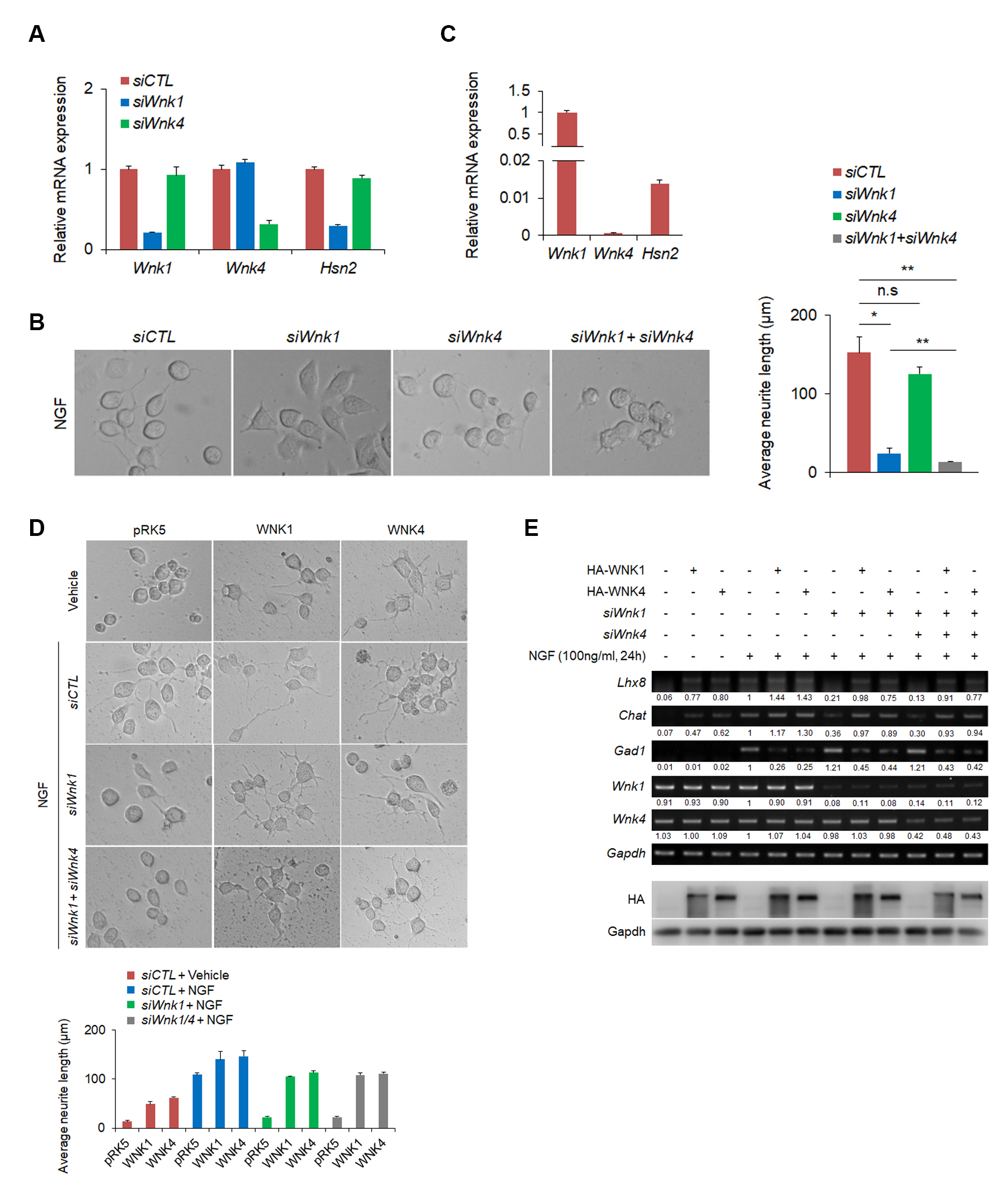


**Supplementary Figure 1.** (A) Expression of mouse *Wnk1*, *Wnk4* and *Hsn2* in *siCTL*-, *siWnk1*- or *siWnk4*-treated Neuro2A cells. (B) Neurite elongation of Neuro2A cells treated with 100 ng/ml NGF-containing FBS-free medium for 24 h. Cells were transiently transfected with control siRNA (siCTL), *siWnk1* and/or *siWnk4.* (C) Endogenous mRNA expression of Wnk1, Wnk4 and Hsn2 were quantified by qPCR. (D) Neurite outgrowth of Neuro2A cells treated with 100 ng/ml NGF-containing FBS-free medium for 24 h. Cells were transiently transfected with indicated vectors and control siRNA (siCTL), *siWnk1* and/or *siWnk4* 24 h prior to treatment. (E) RT-PCR analysis of mRNA levels of *Lhx8*, *ChAT* and *Gad1* in Neuro2A cells indicated in D.


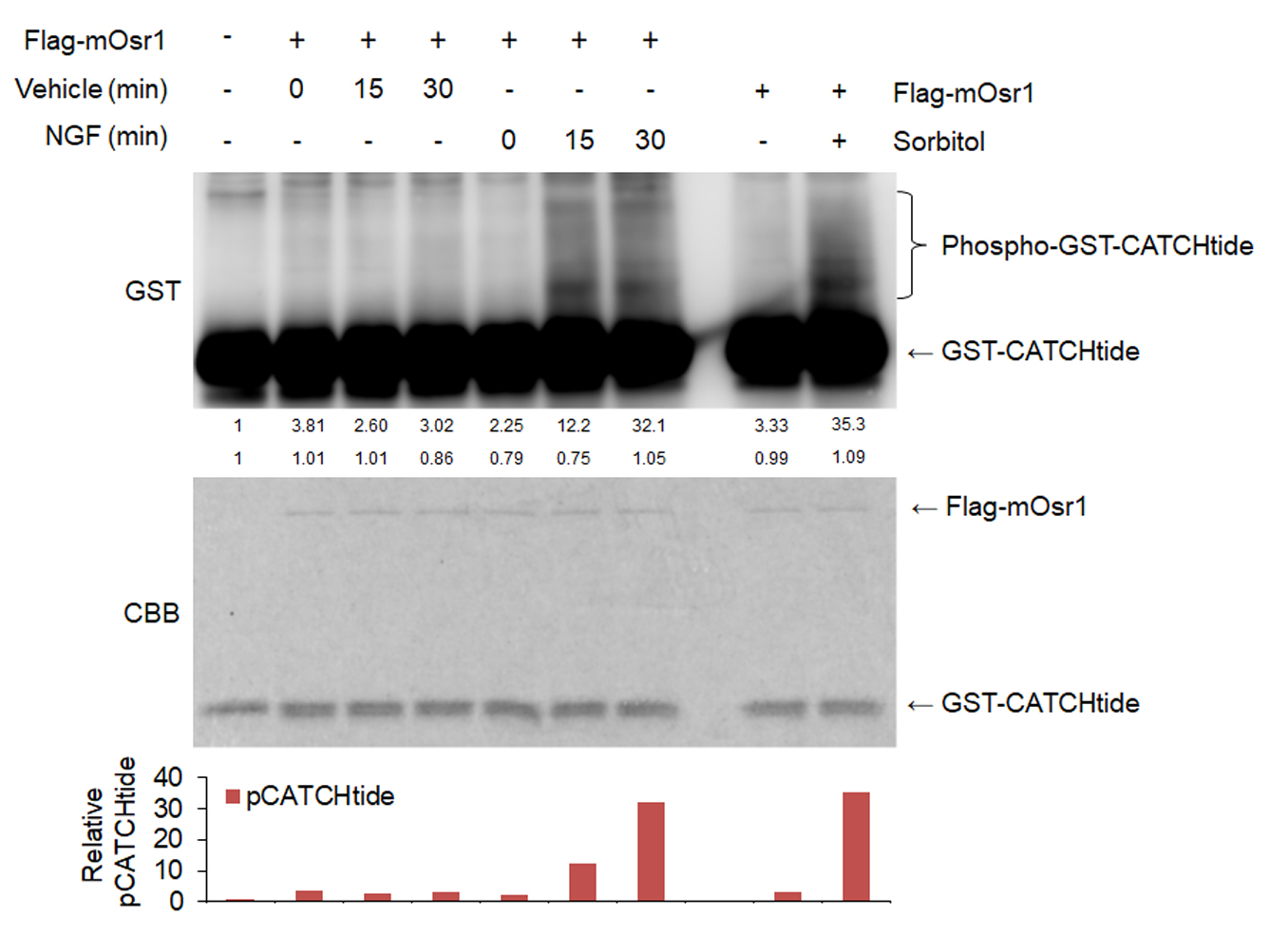


**Supplementary Figure 2.** Phosphorylation of CATCHtide by mOsr1 was analyzed using Phos-tag SDS-PAGE.

**
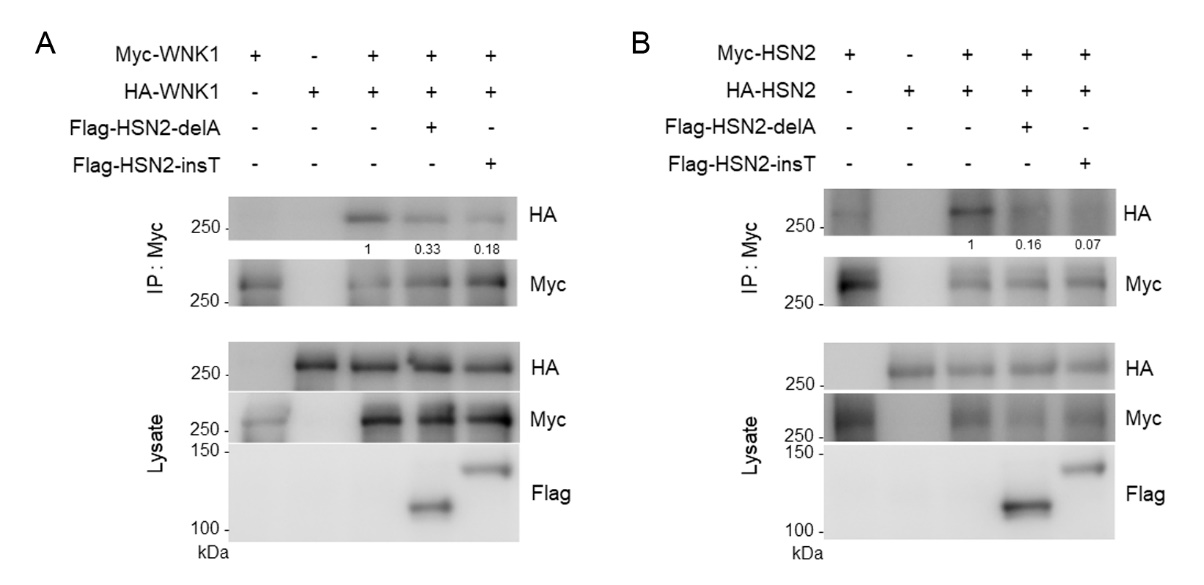
**

**Supplementary Figure 3.** (A and B) The homodimerization of WNK1 (A) or HSN2 (B) with and without HSN2-delA and HSN2-insT mutants was analysed in HEK293T cells by immunoprecipitation assays. Cells were transiently transfected with indicated vectors and 48 h later lysates were immunoprecipitated with a Flag antibody for 4 h. Immunoprecipitates were subjected to immunoblotting assays with the indicated antibodies.

**Supplementary Figure 4.** The original images in all Figures and Supplementary Figures. Almost membranes were cut prior to hybridization with antibodies.


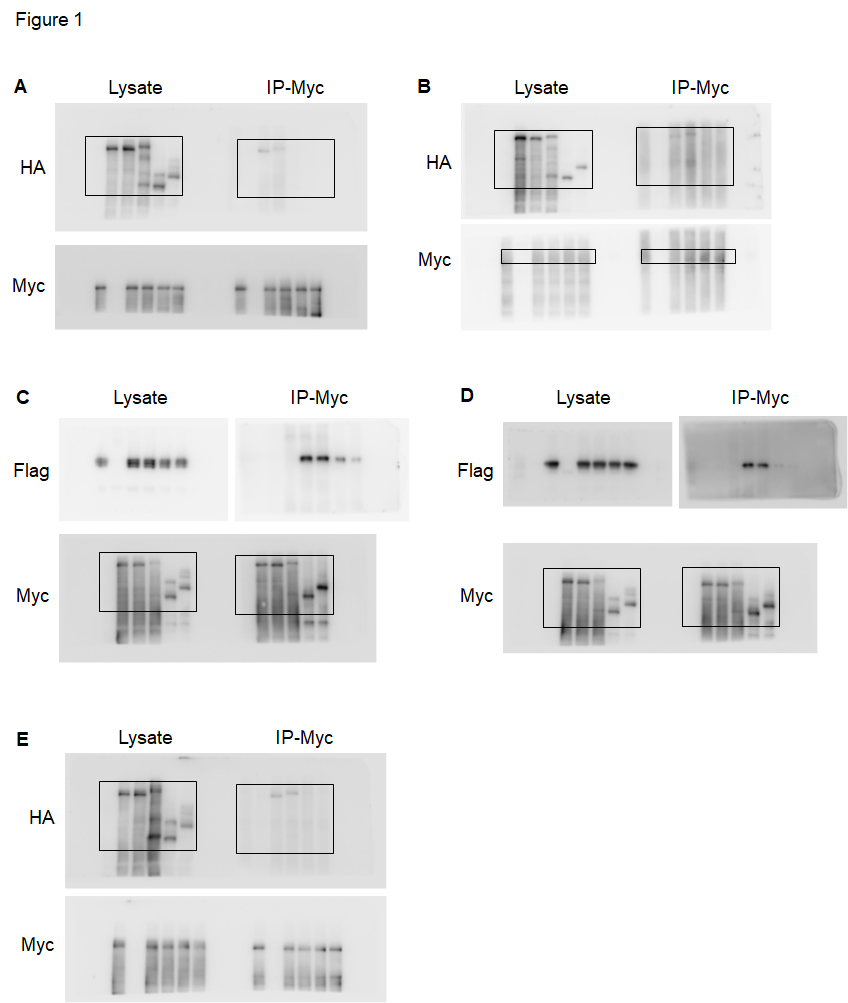


**Supplementary Figure 4 continued**


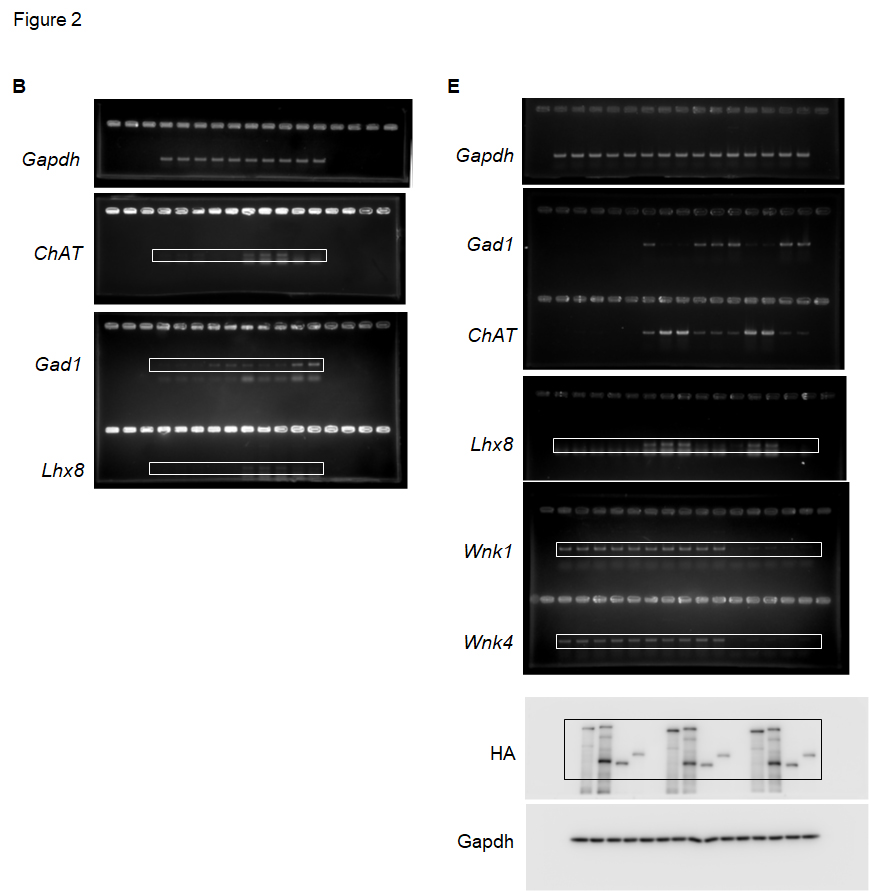


**Supplementary Figure 4 continued**


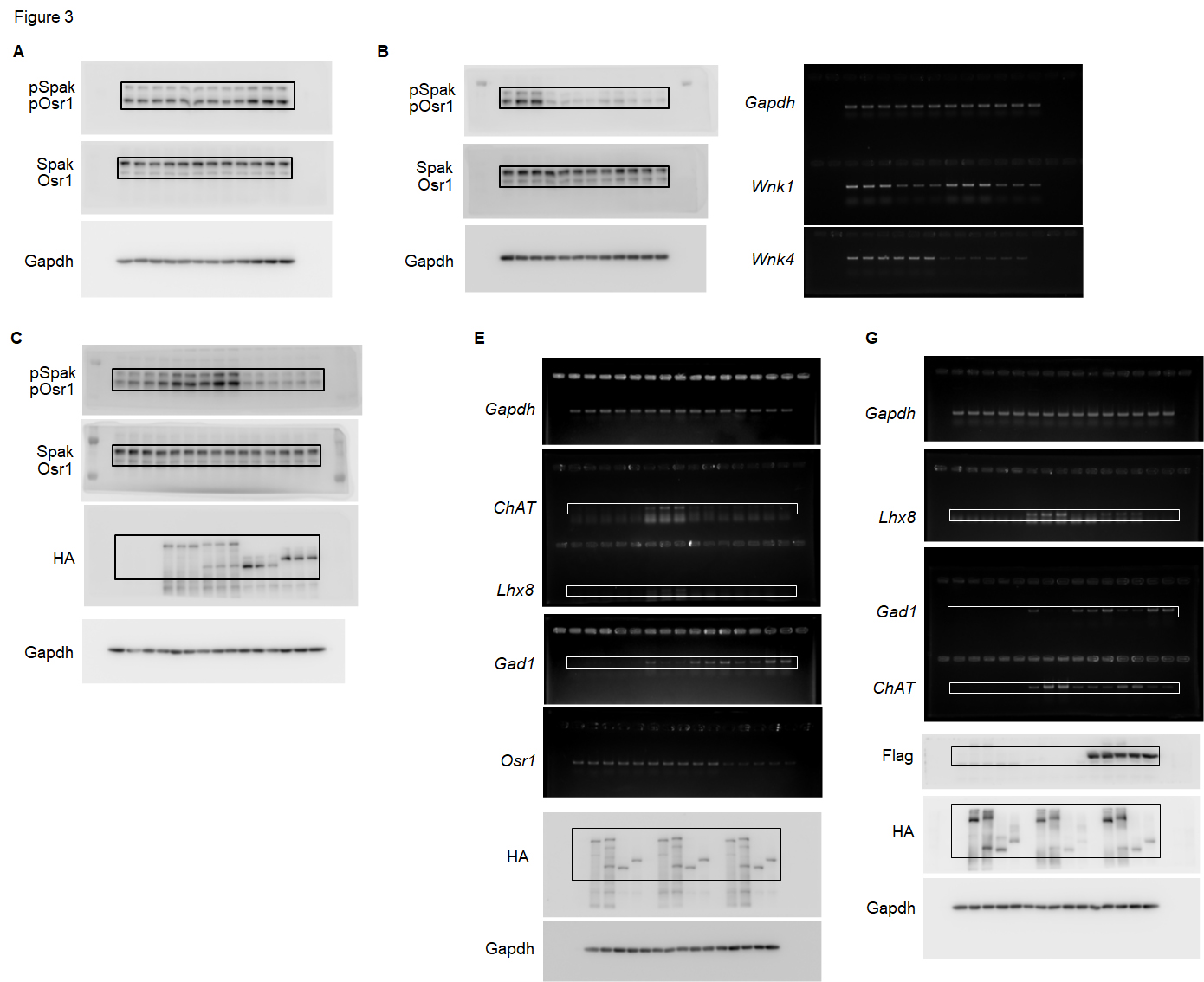


**Supplementary Figure 4 continued**


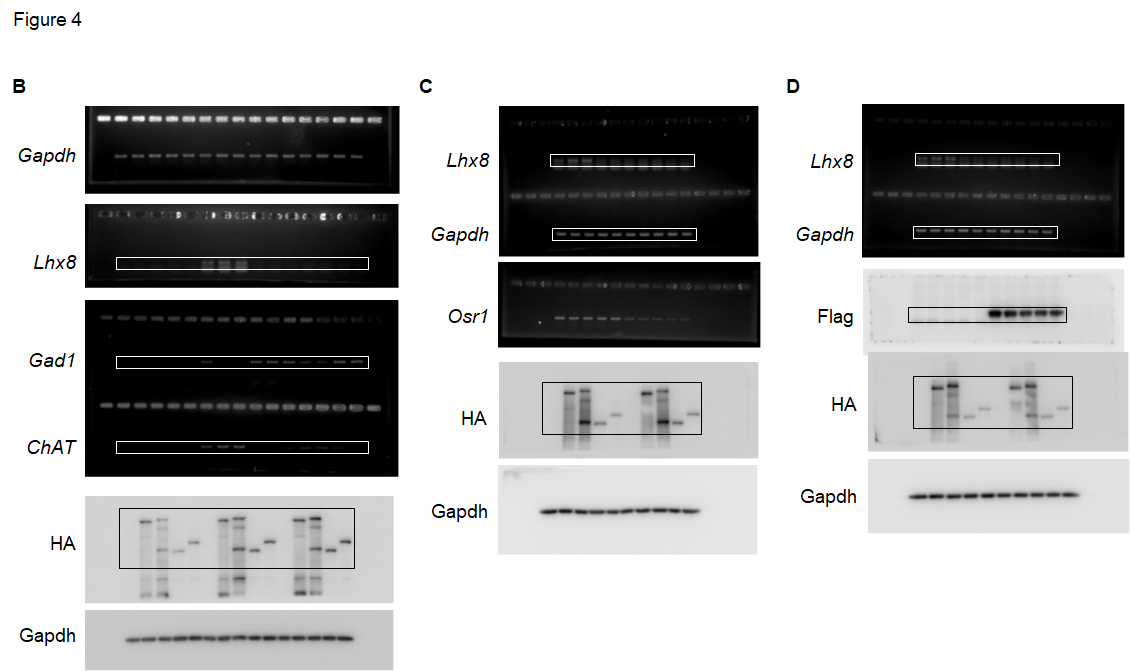


**Supplementary Figure 4 continued**


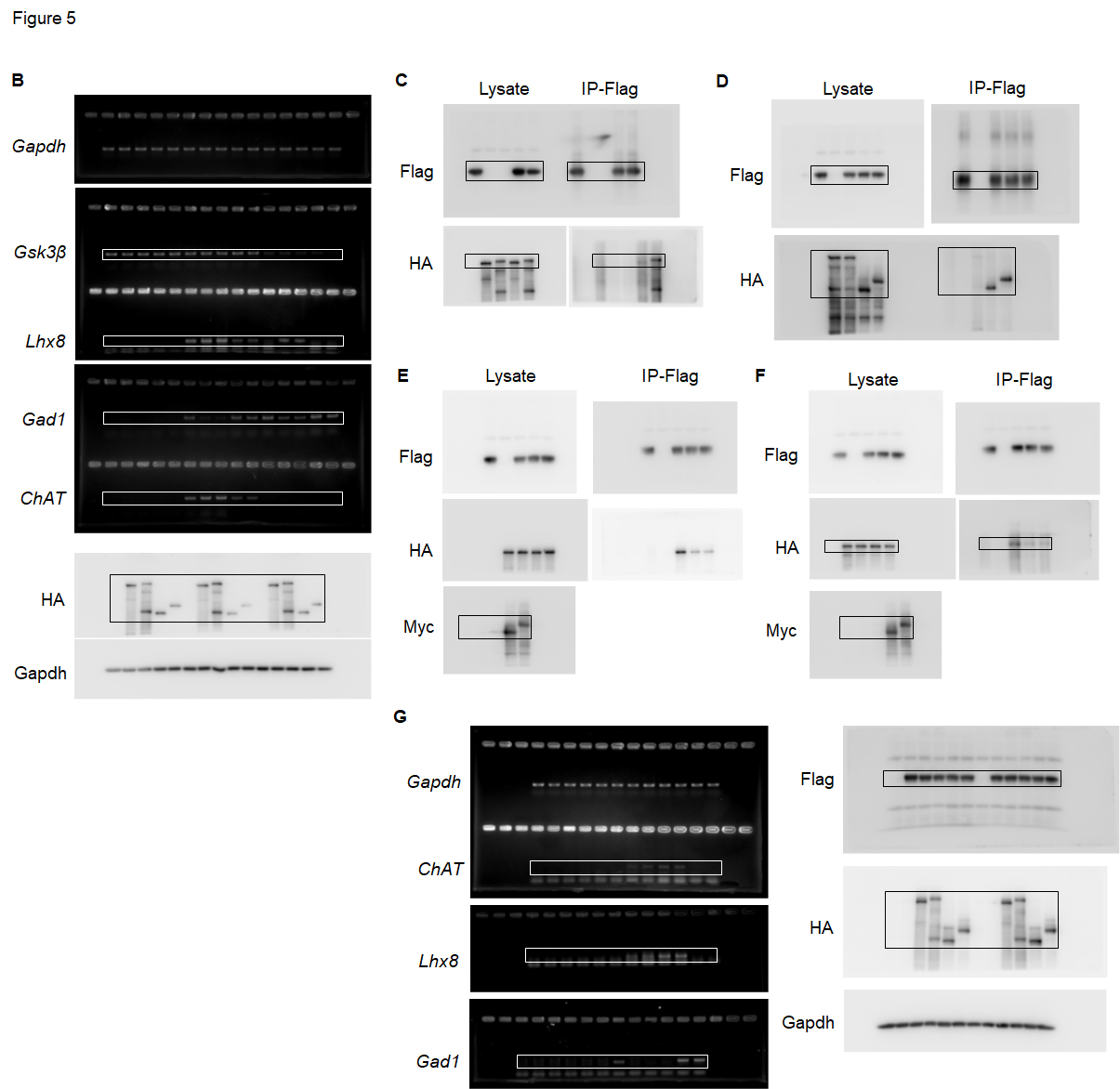


**Supplementary Figure 4 continued**


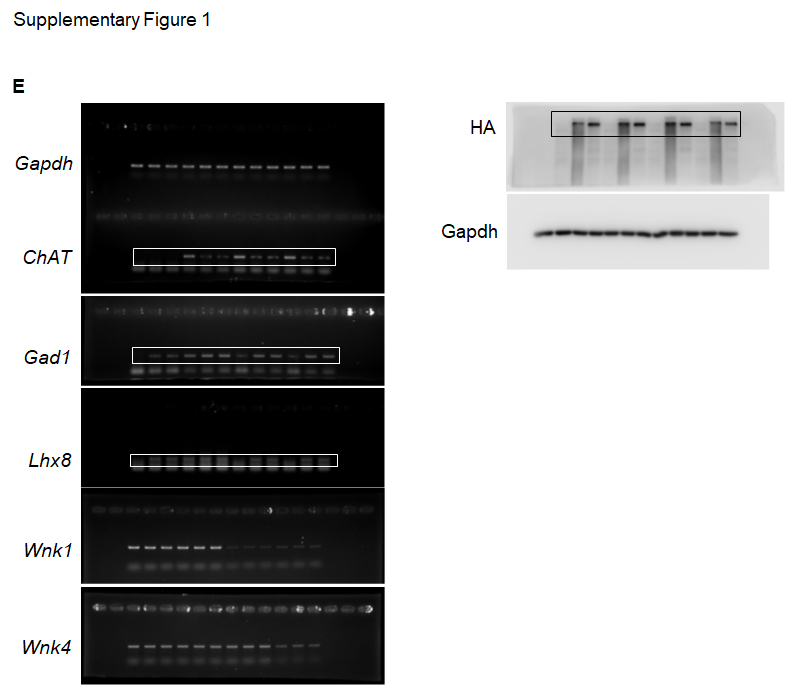


**Supplementary Figure 4 continued**


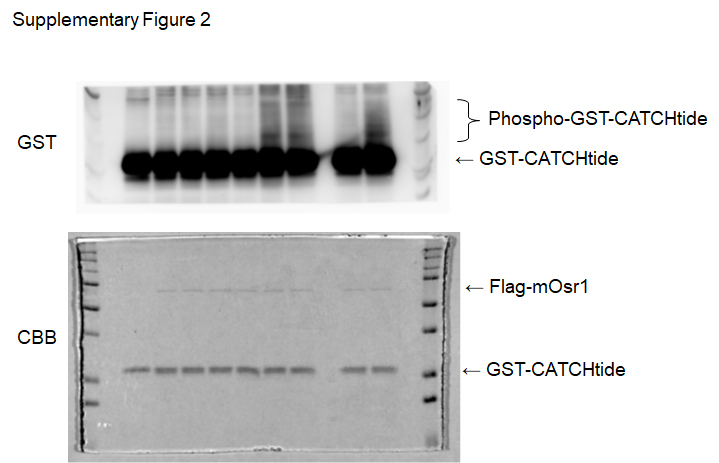


**Supplementary Figure 4 continued**


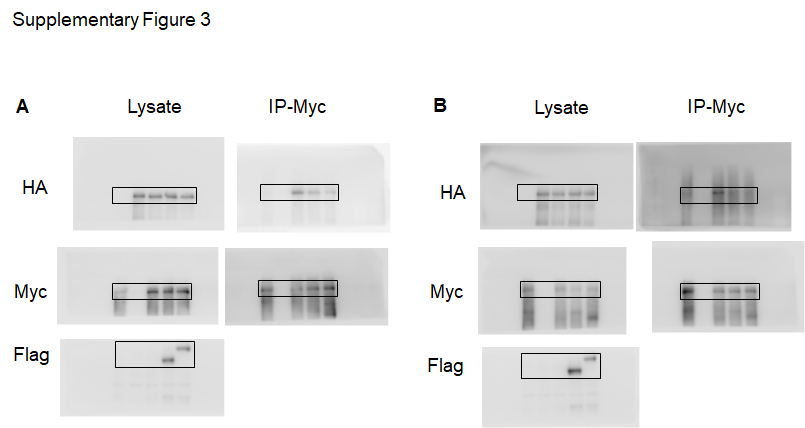

Supplement: Supplementary file 1 — Supplementary Figures. [file 41598_2022_20271_MOESM1_ESM.docx]
